# Malaria profile and socioeconomic predictors among under-five children: an analysis of 11 sub-Saharan African countries

**DOI:** 10.1186/s12936-023-04484-8

**Published:** 2023-02-14

**Authors:** Seun Anjorin, Elvis Okolie, Sanni Yaya

**Affiliations:** 1 Big Data Institute, Nuffield Department of Population Health, University of Oxford, Oxford, UK; 2 School of Health and Life Sciences, Teesside University, Middlesbrough, UK; 3 Department of Public Health, David Umahi Federal University of Health Sciences, Uburu, Ebonyi Nigeria; 4 The George Institute for Global Health, Imperial College London, London, UK; 5 School of International Development and Global Studies, University of Ottawa, 120 University Private, Ottawa, K1N 6N5 Canada

**Keywords:** Malaria, Children, Under-five, Determinants, Sub-Saharan Africa

## Abstract

**Background:**

African region accounts for 95% of all malaria cases and 96% of malaria deaths with under-five children accounting for 80% of all deaths in the region. This study assessed the socioeconomic determinants of malaria prevalence and provide evidence on the socioeconomic profile of malaria infection among under-five children in 11 SSA countries.

**Methods:**

This study used data from the 2010 to 2020 Demographic and Health Survey (DHS). The survey used a two-stage stratified-cluster sampling design based on the sampling frame of the population and housing census of countries included. Statistical analyses relied on Pearson’s χ2, using the CHAID decision-tree algorithm and logistic regression implemented in R V.4.6.

**Results:**

Of 8547 children considered, 24.2% (95% confidence interval CI 23.4–25.05%) had malaria infection. Also, the prevalence of malaria infection seems to increase with age. The following variables are statistically associated with the prevalence of malaria infection among under-five children: under-five child’s age, maternal education, sex of household head, household wealth index, place of residence, and African region where mother–child pair lives. Children whose mothers have secondary education have about 56% lower risk (odds ratio = 0.44; 95% CI 0.40–0.48) of malaria infection and 73% lower (odds ratio = 0.37; 95% CI 0.32–0.43) among children living in the richest households, compared to children living in the poorest households.

**Conclusions:**

The findings of this study provide unique insights on how socioeconomic and demographic variables, especially maternal education level significantly predicts under-five malaria prevalence across the SSA region. Therefore, ensuring that malaria interventions are underpinned by a multisectoral approach that comprehensively tackles the interplay of maternal education and other socioeconomic variables will be critical in attaining malaria prevention and control targets in SSA.

## Background

Increasing knowledge of the health and socioeconomic impact of malaria especially in sub-Saharan Africa (SSA) has led to growing global consensus towards malaria elimination. The last two decades have witnessed an increase in global interventions directed at expanding access to malaria prevention, diagnosis, and treatment especially in SSA countries disproportionately affected by malaria [1–5]. For instance, investments in malaria prevention more than doubled from US$ 960 million to US$ 2.5 billion between 2005 and 2014 [3]. It is estimated that between 2000 and 2020, 1.7 billion malaria cases and 10.6 million malaria deaths were averted [4]. These gains in malaria morbidity and mortality especially in SSA have been associated with expansion in the implementation of effective malaria prevention and control measures including the use of insecticide-treated nets (ITNs), artemisinin-based combination therapy (ACT), and vector control strategies. Despite progress in the scale-up of malaria focused interventions, achieved results have been described as fragile and decline in malaria incidence remain unevenly distributed especially for countries in SSA where malaria burden remains high [3–6].

In 2020, global malaria estimates stood at 241 million cases and 627,000 deaths across 85 malaria-endemic countries with Africa especially SSA countries bearing the greatest burden of the disease [4]. The 2020 estimate shows an increase of 14 million cases from the 2019 Figs. (227 million) and such an increase has been mostly attributed to the negative impact of COVID-19 pandemic on access to malaria services [4, 7]. Regrettably, the African region accounts for 95% of all malaria cases and 96% of malaria deaths with under-five children accounting for 80% of all deaths in the region [4]. Six SSA countries namely Nigeria (27%), the Democratic Republic of Congo (12%), Uganda (5%), Mozambique (4%), Angola (3.4%), and Burkina Faso (3.4%) account for more than half (55%) of all global malaria cases; highlighting malaria as a public health problem in SSA [4]. Such high malaria concentration in SSA reflects the slow progress of interventions to eliminate malaria and has been attributed to an interplay of broader factors such as the lack of robust financing mechanisms, weak regional and national political commitment, poverty, parasite and mosquito resistance, poor health systems, and general health-seeking behaviour [2, 8].

While almost half of the world population especially those in endemic areas are at risk of malaria infection, pregnant women and under-five children in SSA are even at greater risk of malaria-related mortality and morbidity [1, 4, 9, 10]. Malaria is a major cause of under-five mortality in SSA and severe malaria cases remain high among this group [4, 9, 11]. The lack of a fully functional immune system among under-five children means that a case of uncomplicated malaria could rapidly progress to severe malaria and potentially followed by anaemia and death [9, 10]. Additionally, malaria among under-five children could facilitate poor educational outcomes and reduced cognitive function [10, 12, 13]. Previous studies have identified factors such as socioeconomic status including household size and wealth index, access to and use of ITNs, mother/caregiver educational level, child’s age, place of residence, environmental conditions, and access to health services as key determinants of malaria among under-five children in SSA [1, 11, 14–16]. Hence, greater understanding of the complex interactions between these factors is critical to policy and programme efforts towards the elimination of malaria among under-five children and the SSA population in general.

The vision of the Global Technical Strategy for Malaria (2016–2030) to reduce global malaria incidence and mortality by at least 90% in 2030 compared to 2015 renews global focus to end malaria, especially in SSA [8]. Similarly, target 3.2 of the sustainable development goals (SDGs) which calls on countries to reduce preventable deaths among under-five children to at least as low as 25 deaths per 1000 live births highlights the need for additional efforts. In addition, more sophisticated surveillance systems to ensure a relatively accurate picture of malaria burden and the impact of malaria interventions have been advocated [17]. While research on malaria prevalence and efficacy of interventions have received great attention, there is limited knowledge of the socioeconomic profile of under-five children across many SSA countries that are disproportionately affected by malaria. Therefore, this study aims to determine the socioeconomic determinants of malaria prevalence and provide evidence on the socioeconomic profile of malaria infection among under-five children in 11 SSA countries.

## Methods

### Data source

This study used data from the 2010 to 2020 Demographic and Health Survey (DHS). The survey used a two-stage stratified-cluster sampling design based on the sampling frame of the population and housing census of countries included. The final survey unit chosen was the cluster (district or village) and the first stage of sampling involved the selection of clusters known as primary sampling units. The second stage of sampling involved the selection of households from each cluster. Primary sampling units in regions with a very small population were selected with equal size allocation. The DHS data offer a unique opportunity to profile malaria infection in the country selected due to the paucity of routine data, which are associated with unknown denominators and selection bias because all malaria cases are not reported in health facilities. In addition, those data do not include individual and household characteristics considered as predictors of the epidemy. The DHS incorporated five biomarker tests, including malaria testing. Malaria testing was carried out among children aged 6–59 months in half of the 18,360 selected households using microscopy. Using a finger (or heel) prick, a drop of blood was collected on a slide to prepare a thick film. All health technicians were trained to perform finger (or heel) pricks in the field according to the manufacturer’s instructions.

### Site selection

Eleven countries with suitable data for malaria in children and corresponding independent determinants were selected and included in this study. Nevertheless, the 11 countries included in the study covered regions of Africa where malaria is highly endemic. A total of 8,547 children aged 6–59 months were assessed for malaria.

### Study variables

The malaria infection status of an under-five is the dependent variable for this study; it was defined as a binary variable; positive (coded as 1) or negative (coded as 0). The independent variables include 10 variables grouped into 3 major types: child variables (sex, age, living arrangement, whether under-five child slept under an ITN the night preceding data collection); mother’s education and household’s characteristics (sex of the head of household, age of the head of household, wealth index); and contextual factors including place of residence (if rural or urban) and African region. These variable selections were informed by previous studies on factors associated with malaria infection [18–24].

### Statistical analyses

Statistical analyses relied on Pearson’s χ2, using the CHAID decision-tree algorithm implemented in R V.4.6, and logistic regression. Pearson’s χ2 was performed to identify associations between the malaria infection (positive or negative) and independent variables, including socioeconomic and demographic characteristics. The study also employed logistic regression to identify predictors of malaria infection among under-five children. This consists of comparing the proportions using the odds ratio. For each selected category, the model estimates the parameter ß (the ratio between the logit of a selected group and that of the reference group) and calculates the odds ratios while specifying their significance level (95% in this case). If the odds ratio is equal to one, there is no difference between the considered group and the reference group regarding the risk of malaria infection. If the odds ratio is less than unity, children in the considered group are less likely to suffer from malaria infection, compared to children in the reference group. In contrast, if the odds ratio is greater than one, children in the selected group are more likely to suffer from malaria infection than children in the reference group. However, the logistic regression model fails to incorporate non-monotonic relationships. Furthermore, it does not automatically detect interactions between segments or categories of independent variables. Significant differences have been established at p < 0.05.

As further analysis, the study applied the nominal CHAID model to identify the most significant determinants of malaria infection among under-five children and to describe the characteristics of the most-at-risk children for malaria infection considering interactions between predictors [25–27]. The model operates sequentially by recursively splitting under-five children into separate and distinct segments called nodes. The variation of the prevalence of malaria infection is minimized within each node and is maximized between nodes. After the initial splitting of the under-five children who received a malaria test into different nodes based on the most significant predictor, the model repeats the process on each of the nodes until no significant predictors remain or until the number of observations in the node does not allow further partitions.

The maximum partitioning was limited to 1000 because of the sample size. Ideally, the minimum number of cases is estimated at 50 cases for child nodes, although the minimum number of cases can be lowered [26–28]. CHAID displays outcomes in a hierarchical tree-structured form, in which the root is the population, which is under-five children who received a malaria test in this case. The root node, ‘Node 0’ or ‘initial segment,’ is the outcome variable, and subsequent levels include the parent node and child node. The parent node is the upper node compared with nodes on the subsequent (lower) level, whereas any sub-node of a given node is called a child node. Sibling nodes are nodes on the same hierarchical level under the same parent node. Ancestor nodes comprise all nodes higher than a given node in the same lineage, and all nodes below the given node are called descendants. The terminal nodes are any node that does not have child nodes. They are the last categories of the CHAID tree. Findings include a table with five major columns describing each terminal node regarding content, population size, number with malaria infection, and the prevalence of malaria infection. The analysis is focused on column 4, the prevalence of malaria infection in each terminal node (category).

### Data analysis

Data were weighted for CHAID and applied the SVY for the logistic regression in R v4.6 to account for the complex design of the household survey. Missing values were treated as a separate category. For instance, the “Do not know” category for mother’s education included children whose mother’s education was missing.

### Ethical considerations

The DHS questionnaire, procedures, and testing protocol underwent each host country ethical review and were reviewed by ICF institutional review board. Details of this has been published elsewhere [29]. Participation in the individual survey and malaria testing was voluntary, and parents signed the consent form before the interview and before their child’s blood collection. Interviews and biomarker testing were performed as privately as possible. Results of interviews and biomarker testing were strictly confidential. Only the DHS research team (interviewers, health specialists, editors, and supervisors) were allowed to access the data, essentially for communications. Each respondent’s interview and biomarker data files were identified only by a series of numbers.

## Results

### Socio-economic and demographic characteristics of under-five children

Table 1 shows the distribution of the respondents included in the study by selected background characteristics and country. Of the total 72,999 children aged 6–59 months who were tested for malaria, 41.3% were female. The distribution of the sample by age shows that 11.4% of the population was aged 6–11 months. A majority of participants lived in rural areas (71.5%) and households headed by males (80.5%). More than 60% of the participants were living in households headed by people aged 35 years and above, and 4.1% were living in households headed by people aged < 25 years. More than half of children assessed (59.4%) slept under an ITN the night preceding the survey and 22.67% of children were living in the poorest households, and 17% were living in the richest households.

**Table 1:** Descriptive statistics with the pooled sample of DHS data from 11 sub-Sahara Africa

Independent Variables	Overall	Negative	Positive	p test
	72999	55479	17520	
Child’s age (%)				< 0.001
6–11	6908 (11.4)	5563 (12.5)	1345 (8.2)	
12–23	13,701 (22.6)	10,624 (23.9)	3077 (18.9)	
24–35	13,342 (22.0)	9775 (22.0)	3567 (21.9)	
36–47	13,410 (22.1)	9328 (21.0)	4082 (25.0)	
48–59	13,348 (22.0)	9109 (20.5)	4239 (26.0)	
Child is female (%)	30,152 (41.3)	22,118 (39.9)	8034 (45.9)	< 0.001
Child slept under ITN = Yes (%)	40,325 (59.4)	30,354 (59.7)	9971 (58.4)	0.002
Maternal education (%)				< 0.001
No education	23,522 (42.1)	14,315 (35.0)	9207 (61.5)	
Primary	19,894 (35.6)	15,831 (38.7)	4063 (27.2)	
Secondary and higher	12,432 (22.3)	10,739 (26.3)	1693 (11.3)	
Female household head (%)	14,932 (20.5)	12,165 (21.9)	2767 (15.8)	< 0.001
Household head age (%)				0.002
< 25	3003 (4.1)	2281 (4.1)	722 (4.1)	
25–34	21,174 (29.0)	16,077 (29.0)	5097 (29.1)	
35–44	22,946 (31.4)	17,626 (31.8)	5320 (30.4)	
45-max	25,872 (35.4)	19,491 (35.1)	6381 (36.4)	
Household wealth (%)				< 0.001
Poorest	16504 (22.6)	11088 (20.0)	5416 (30.9)	
Poorer	15157 (20.8)	10612 (19.1)	4545 (25.9)	
Middle	14682 (20.1)	10862 (19.6)	3820 (21.8)	
Richer	14240 (19.5)	11587 (20.9)	2653 (15.1)	
Richest	12416 (17.0)	11330 (20.4)	1086 (6.2)	
Residence = rural (%)	52175 (71.5)	37818 (68.2)	14357 (81.9)	< 0.001
Region (%)				< 0.001
Central Africa	8186 (11.2)	6030 (10.9)	2156 (12.3)	
Eastern Africa	33013 (45.2)	29179 (52.6)	3834 (21.9)	
Western Africa	31800 (43.6)	20270 (36.5)	11530 (65.8)	
Country (%)				< 0.001
Benin	5956 (8.2)	3583 (6.5)	2373 (13.5)	
Burkina Faso	6102 (8.4)	2138 (3.9)	3964 (22.6)	
Burundi	6924 (9.5)	5143 (9.3)	1781 (10.2)	
Congo Democratic Republic	8186 (11.2)	6030 (10.9)	2156 (12.3)	
Cote d’Ivoire	4513 (6.2)	3775 (6.8)	738 (4.2)	
Ghana	3197 (4.4)	2215 (4.0)	982 (5.6)	
Mozambique	4898 (6.7)	3433 (6.2)	1465 (8.4)	
Nigeria	8144 (11.2)	6208 (11.2)	1936 (11.1)	
Rwanda	11051 (15.1)	10981 (19.8)	70 (0.4)	
Tanzania	10140 (13.9)	9622 (17.3)	518 (3.0)	
Togo	3888 (5.3)	2351 (4.2)	1537 (8.8)	

### Bivariate analysis: factors associated with malaria prevalence

Overall, out of 8547 children considered, 24.2% (95% confidence interval CI 23.4%-25.05%) had malaria infection. Also, the prevalence of malaria infection seems to increase with age. The percentage of children with malaria was estimated at 8.2% among children aged 6–11 months and 26% among those aged 48–59 months. The prevalence of malaria infection was low among children living with their mothers alone (23%) or living with both parents (25%), compared to children living with others (31%). Table 2 also shows a significant negative association between mother’s education and malaria infection among under-five children. It was higher among children whose mother did not attend school (61.5%) and lower among children whose mother had secondary or higher education (11.3%).

**Table 2:** Multivariate logistic regression result of risk of malaria infection among under-five children in 11 African countries

	Model 1		Model II		Model III		Model IV	
Survey Year								
2010–2015	1.00	[1.00,1.00]	1.00	[1.00,1.00]	1.00	[1.00,1.00]	1.00	[1.00,1.00]
2016–2020	0.43^***^	[0.40,0.47]	0.43^***^	[0.40,0.47]	0.44^***^	[0.40,0.49]	0.46^***^	[0.42,0.50]
Child’s age (%)								
6–11	1.00	[1.00,1.00]					1.00	[1.00,1.00]
12–23	1.23^***^	[1.12,1.34]					1.27^***^	[1.16,1.40]
24–35	1.51^***^	[1.39,1.64]					1.61^***^	[1.47,1.76]
36–47	1.80^***^	[1.66,1.96]					1.86^***^	[1.70,2.04]
48–59	1.94^***^	[1.79,2.11]					2.02^***^	[1.85,2.21]
Gender								
Male	1.00	[1.00,1.00]						
Female	0.98	[0.94,1.02]						
Child slept under ITN								
No	1.00	[1.00,1.00]					1.00	[1.00,1.00]
Yes	1.01	[0.94,1.07]					0.97	[0.91,1.04]
Maternal education								
No education			1.00	[1.00,1.00]			1.00	[1.00,1.00]
Primary			0.45^***^	[0.42,0.48]			0.64^***^	[0.60,0.69]
Secondary and higher			0.34^***^	[0.31,0.38]			0.44^***^	[0.40,0.48]
Female household head								
No			1.00	[1.00,1.00]			1.00	[1.00,1.00]
Yes			0.78^***^	[0.72,0.84]			0.89^**^	[0.82,0.96]
Household head age								
< 25			1.00	[1.00,1.00]				
25–34			0.96	[0.84,1.08]				
35–44			0.93	[0.82,1.06]				
45-max			1.04	[0.91,1.18]				
Household wealth (%)								
Poorest			1.00	[1.00,1.00]			1.00	[1.00,1.00]
Poorer			0.95	[0.87,1.03]			0.91^*^	[0.84,0.99]
Middle			0.84^***^	[0.77,0.92]			0.82^***^	[0.74,0.89]
Richer			0.60^***^	[0.54,0.66]			0.64^***^	[0.57,0.71]
Richest			0.31^***^	[0.27,0.35]			0.37^***^	[0.32,0.43]
Residence								
Urban					1.00	[1.00,1.00]	1.00	[1.00,1.00]
Rural					3.20^***^	[2.90,3.53]	1.74^***^	[1.56,1.95]
Region (%)								
Central Africa					1.00	[1.00,1.00]	1.00	[1.00,1.00]
Eastern Africa					1.08	[0.89,1.31]	0.94	[0.77,1.14]
Western Africa					3.22^***^	[2.89,3.58]	2.45^***^	[2.20,2.72]
N	72,999		72,999		72,999		72,999	

The prevalence of malaria infection was higher in rural areas (81.9%); it also varied widely size varied by country, from 0.4% in Rwanda to 22.6% (Burkina Faso). The proportion of children with malaria was lower in the richest households (6.2%), compared to those living in other households. However, the result shows that the prevalence of malaria infection among children who slept under an ITN (58.7) the previous night is almost the same compared to those who did not sleep under an ITN (59.7%). As shown in Table 1, all the independent variables included in the bivariate analysis were statistically significantly associated with malaria infection status.

### Socioeconomic predictors of malaria: findings from the logistic regression model

Of the nine independent variables included in the logistic regression model as shown in Table 2, only six of them are statistically associated with the prevalence of malaria infection among under-five children in the 11 SSA: under-five child’s age, maternal education, sex of household head, household wealth index, place of residence, and African region where mother–child pair lives. The risk of malaria infection increases significantly with a child’s age. Compared to children aged 6–11 months, those aged 12–23 months have 1.27 times more risk of malaria (95% CI = 1.16–1.40). This risk is estimated at 1.61 (95% CI = 1.47–1.76) for children aged 24–35 months, 1.86 (95% CI = 1.76–2.04) for children aged 36–47 months, and 2.02 (95% CI = 1.85–2.21) for children aged 48–59 months. Also, the likelihood of malaria infection is lowest among under-five children living in wealthy households. The risk of malaria infection is 36% lower (odds ratio = 0.64; 95% CI = 0.57–0.71) among children living in richer households, and about 73% lower (odds ratio = 0.37; 95% CI = 0.32–0.43) among children living in the richest households, compared to children living in the poorest households.

Considering mother’s education, children whose mothers have secondary education have about 56% lower risk (odds ratio = 0.44; 95% CI = 0.40–0.48) of malaria infection, compared to those whose mothers did not attend school. Under-five children of mothers with primary school education alone were 36% less likely to have malaria infection (odds ratio = 0.64; 95% CI = 0.60–0.69). After controlling for other variables, the risk of malaria infection is highest among under-five living in western African countries when compared to those living in Central African countries. There was no significant difference among under-five children living in Eastern and Central African countries.

### Socioeconomic predictors of malaria: findings from the CHAID model

Table 3 shows summary details of the specifications used to build the final CHAID model. The five variables that were statistically significant from the multivariate logistic regression were added to the final model. Based on literature consistency on ITN, we included the usage of ITN the night before the survey as part of the variable.

**Table 3:** Malaria prevalence among under-five children: Summary of CHAID model

Model components	Model specification	Results
Dependent variable	Parasitaemia (via microscopy) in children aged 6–59 Months	25%
Independent variables	Child’s age, ITN used the night before the survey, place of residence, mother’s education, wealth index and region	Province, child’s age, place of residence, mother’s education, wealth index
Maximum tree depth	3	4
Minimum number of children in parent node	1000	1000
Minimum number of children in child node	200	200
Number of nodes	Na	25
Number of terminal nodes	Na	40

**Fig. 1:**
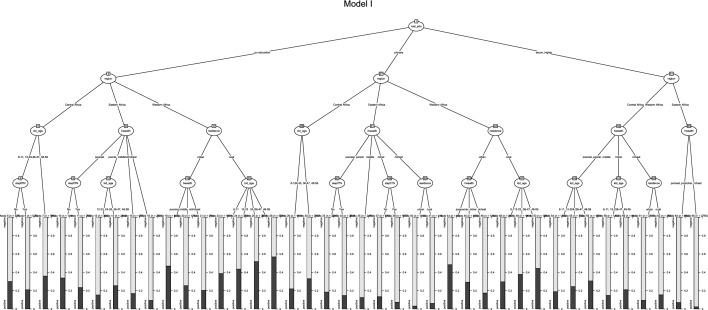
CHAID model for socioeconomic predictors of malaria

The CHAID model splits participants into 26 homogeneous sub-groups, or terminal nodes, regarding the prevalence of malaria infection. The CHAID tree diagram shown in Fig. 1 shows that maternal educational attainment (χ2 = 577.64, p < 0.001) is the best predictor of malaria infection. Table 4 was used to simplify the result from the CHAID tree in Table 1; it shows the summary of 1st to 4th level predictors of malaria infection among under-five children in 11 SSA countries by maternal education attainment.

**Table 4:** Summary of Socioeconomic predictors of malaria infection among under-five children by maternal education attainments

First level predictors	Second & third level predictor	Fourth level predictors
No education	*Region* Central & kid age (χ2 = 29.13.64, p < 0.001) Eastern & household wealth index (χ2 = 100.07.6, p < 0.001) Western & place of residence (χ2 = 474.04, p < 0.001)	Used ITN the night before the survey (χ2 depends on maternal education and child’s age) Child’s age (χ2 depends on maternal education and household wealth index) Household wealth index (χ2 depends on maternal education and lace of residence)
Primary education	*Region* Central & kid age (χ2 = 28.64, p < 0.001) Eastern & household wealth index (χ2 = 195.6, p < 0.001) Western & place of residence (χ2 = 183.25, p < 0.001)	Place of residence among the richest, else, child’s age, else, usage of ITN the night before the survey (χ2 depends on maternal education and household wealth index) In urban, household wealth index; in rural, child’s age (χ2 depends on maternal education and lace of residence)
Secondary education and higher	*Region* Central & household wealth index (χ2 = 41.21, p < 0.001) Eastern & household wealth index (χ2 = 40.39.6, p < 0.001) Western & household wealth index (χ2 = 226.32.6, p < 0.001)	Place of residence among the richest, else, child’s age The same as Central African region

Depending on the maternal educational attainment and region of SSA, the 3rd and 4th level predictors include child’s age, place of residence and wealth index. No 4th level malaria infection predictor was identified among children of women with primary education living in Central African countries. Among children whose mothers have secondary education and above, the household wealth index is the 3rd level predictor irrespective of the region of residence. Nevertheless, place of residence (rural or urban) differentiates this for the richest while it differs by child’s age for those in poorest, poorer, and average household income.

## Discussion

Malaria among under-five children is an important public health problem and a key barrier to achieving the SDGs in SSA. This study examined the socioeconomic determinants of malaria prevalence and provide evidence on the socioeconomic profile of malaria infection among under-five children in 11 SSA countries using Chi-square, logistic regression, and the CHAID model. Due to the multi-country approach of this study, findings from the CHAID model which allowed for the identification of the most significant determinants of malaria infection among under-five children and interactions between predictors across regions will form a substantial part of this discussion.

Overall, this study found a 24.2% malaria prevalence among under-five children across the 11 SSA countries. For the bivariate analysis (Chi-square), findings show that all the independent variables (survey year, under-five child’s age, gender, child slept under ITN, maternal education, female household-head, household-head age household wealth index, place of residence, and African region where mother–child pair lives) were significantly associated with malaria infection status among under-five children (Table 1). The logistic regression model in this study revealed that six out of the nine included independent variables (under-five child’s age, maternal education, female of household head, household wealth index, place of residence, and African region where mother–child pair lives) had statistically significant associations with malaria prevalence among under-five children assessed. This finding is similar to those documented by previous studies conducted in the SSA region [1, 11, 16, 30].

The results from the CHAID model in this study show maternal educational attainment as the best predictor of malaria infection among under-five children in the 11 SSA countries. This finding reinforces the results of the Chi-square and logistic regression analysis. For instance, a negative association was found between malaria infection and mothers’ education as malaria prevalence was higher among children whose mothers did not attend school (61.5%) compared to those whose mothers had secondary or higher education (11.3%). This finding agrees with previous studies that reported a similar association between maternal education and malaria prevalence among children [16, 30, 31]. Low educational status among mothers may facilitate poor knowledge of malaria prevention, risk factors, and symptoms leading to higher malaria prevalence among their children [9, 16]. Additionally, such poor knowledge may translate to delay in seeking healthcare and facilitate late detection of malaria infection among under-five children and increased risk of complications [1, 30]. In contrast, mothers with higher levels of education are likely to have better agency in preventing malaria among their children and seeking healthcare services early [31]. Policy and programme initiatives targeted at improving the education of women and girls will be vital in reducing the burden of malaria among children in the SSA region [16, 31].

Based on the different maternal education levels, the CHAID model revealed other variables that predicted malaria infection profile by region (Central, Eastern, and Western). For mothers with no education or primary education, significant predictors include child’s age (Central), household wealth index (Eastern), and place of residence (Western). However, for mothers with secondary education and higher, household wealth index was the key predictor of malaria infection among children across all the regions. These variables were also significant in other statistical analyses employed in this study.

Malaria prevalence among under-five children seemed to increase with age in this study as children within 6–11 months accounted for 8.2% compared to 26% for those within 48–59 months; malaria infection risk was also higher with increasing age. This finding is well documented by previous studies [9, 11, 16, 30]. It is has been suggested that acquired antibodies during pregnancy and breastfeeding may be responsible for a lower prevalence of malaria among infants compared to the older children group [15, 30]. Similarly, infants may be more prioritised leading to greater efforts in ensuring they sleep under an ITN or are well covered compared to older children [16, 30]. Nevertheless, there is a need for future studies to critically investigate the relationship between malaria infection and the age of children in SSA. Such future studies are expected to provide empirical evidence on how different variables interact to mediate or moderate malaria infection across different age categories of children in SSA.

The result of this study demonstrates that place of residence influences malaria infection among under-five children, especially for mothers with no/primary education in the Western SSA region. Children in rural areas accounted for 81.9% of malaria prevalence in this study. Malaria has been referred to as a disease of poverty that disproportionately affects poor people residing in rural settings [15]. In addition to socioeconomic differences, urban residents have better access to health services while rural residents face greater barriers in accessing health services—cost, distance, poor health-seeking behaviour, and availability of health workers [30, 32]. Furthermore, environmental factors such as wetlands and farming activities may promote mosquito breeding which increases the risk of infection among children in rural areas [33].

The significance of household wealth index on malaria infection among under-five children is well published [11, 14, 16, 30] and was observed in this study. In terms of household wealth index, the risk of malaria among children living in the richest households was 73% lower compared to those living in the poorest households. Richer households are likely to be more educated, possess better knowledge on malaria prevention, and have better access to healthcare [16, 30]. Similarly, richer households may have housing with better standards compared to poorer households whose housing standards may increase children’s risk of malaria infection [11, 34]. The impact of household wealth index on malaria prevalence among under-five children in SSA points to the need for malaria prevention and control interventions to adopt a comprehensive approach by moving beyond the sole focus on malaria to incorporating wide-ranging initiatives to improve the socioeconomic status of households [11].

### Strengths and limitations

This study used data from reliable data source—DHS with though sampling strategy and large sample size. However, the study is without limitation as cross-sectional survey design, causality cannot be established. Also, this study did not adjust for seasonal nature of malarian transmission.

## Conclusion

The findings from this study broaden knowledge on the socioeconomic predictors and profile of malaria prevalence among under-five children in SSA through a multi-country perspective. Particularly, results of the multivariate analysis using the CHAID model showed unique insights on how socioeconomic and demographic variables, especially maternal education level significantly predicts under-five malaria prevalence across SSA regions. Based on maternal education level, which was the best predictor across the regions, other predictors such as child’s age, place of residence, and household wealth index were found to be important. This study provides evidence for policymakers and public health actors to consider in policy design and programme implementation activities towards meeting global malaria and related SDG targets in SSA by 2030. While strategic efforts to improve the education of women and girls must be prioritized, ensuring that malaria interventions are underpinned by a multisectoral approach that comprehensively tackles the interplay of other socioeconomic variables will be critical in attaining malaria prevention and control targets in SSA.

## Data Availability

Data for this study were sourced from Demographic and Health surveys (DHS) and available here: http://dhsprogram.com/data/available-datasets.cfm.

## Abbreviations

CHAID: Chi-square automatic interaction detection
DHS: Demographic and health surveys
SSA: Sub-Saharan Africa

## References

[1] Obasohan PE, Walters SJ, Jacques R, Khatab K (2021). A scoping review of selected studies on predictor variables associated with the malaria status among children under five years in sub-Saharan Africa. Int J Environ Res Public Health.

[2] O’Meara WP, Mangeni JN, Steketee R, Greenwood B (2010). Changes in the burden of malaria in sub-Saharan Africa. Lancet Infect Dis.

[3] Cibulskis RE, Alonso P, Aponte J, Aregawi M, Barrette A, Bergeron L (2016). Malaria: global progress 2000–2015 and future challenges. Infect Dis Poverty.

[4] WHO. World malaria report. Geneva: World Health Organization 2021. https://apps.who.int/iris/handle/10665/350147.

[5] Tanner M, Greenwood B, Whitty CJM, Ansah EK, Price RN, Dondorp AM (2015). Malaria eradication and elimination: views on how to translate a vision into reality. BMC Med.

[6] Yang D, He Y, Wu B, Deng Y, Li M, Yang Q (2020). Drinking water and sanitation conditions are associated with the risk of malaria among children under five years old in sub-Saharan Africa: a logistic regression model analysis of national survey data. J Adv Res.

[7] WHO. Fact sheet about malaria. Geneva: World Health Organization 2021. https://www.who.int/news-room/fact-sheets/detail/malaria.

[8] WHO. Global technical strategy for malaria 2016–2030. Copenhagen World Health Organization. Regional Office for Europe. 2015. https://apps.who.int/iris/handle/10665/333980.

[9] Anumudu CI, Okafor CM, Ngwumohaike V, Afolabi K, Nwuba RI, Nwagwu M (2007). Epidemiological factors that promote the development of severe malaria anaemia in children in Ibadan. Afr Health Sci.

[10] Cohee LM, Opondo C, Clarke SE, Halliday KE, Cano J, Shipper AG (2020). Preventive malaria treatment among school-aged children in sub-Saharan Africa: a systematic review and meta-analyses. Lancet Glob Health.

[11] Habyarimana F, Ramroop S (2020). Prevalence and risk factors associated with malaria among children aged six months to 14 years old in Rwanda: evidence from 2017 Rwanda malaria indicator survey. Int J Environ Res Public Health.

[12] Fernando D, Silva DD, Carter R, Mendis KN, Wickremasinghe R (2006). A randomized, double-blind, placebo-controlled, clinical trial of the impact of malaria prevention on the educational attainment of school children. Am J Trop Med Hyg.

[13] Murphy SC, Breman JG (2001). Gaps in the childhood malaria burden in Africa: cerebral malaria neurological sequelae, anemia, respiratory distress, hypoglycaemia and complications of pregnancy The intolerable burden of malaria: a new look at the numbers. Am J Trop Med Hyg.

[14] Rudasingwa G, Cho SI (2020). Determinants of the persistence of malaria in Rwanda. Malar J.

[15] Nyarko SH, Cobblah A (2014). Sociodemographic determinants of malaria among under-five children in Ghana. Malar Res Treat.

[16] Emina JBO, Doctor HV, Yé Y (2021). Profiling malaria infection among under-five children in the democratic Republic of Congo. PLoS ONE.

[17] Snow RW (2014). Sixty years trying to define the malaria burden in Africa: have we made any progress?. BMC Med.

[18] Ferrari G, Ntuku HMT, Ross A, Schmidlin S, Kalemwa DM, Tshefu AK (2016). Identifying risk factors for *Plasmodium* infection and anaemia in kinshasa democratic Republic Congo. Malar J.

[19] Ngatu NR, Kanbara S, Renzaho A, Wumba R, Mbelambela EP, Muchanga SMJ (2019). Environmental and sociodemographic factors associated with household malaria burden in the Congo. Malar J.

[20] Levitz L, Janko M, Mwandagalirwa K, Thwai KL, Likwela JL, Tshefu AK (2018). Effect of individual and community-level bed net usage on malaria prevalence among under-fives in the Democratic Republic of Congo. Malar J.

[21] Report on the Malaria Conference in Equatorial Africa, held under the joint auspices of the World Health Organization and the Commission for Technical Co-operation in Africa South of the Sahara, Kampala, Uganda 27 November-9 December 1950 1951. https://apps.who.int/iris/handle/10665/40153 14913972

[22] Yé Y, Hoshen M, Louis V, Séraphin S, Traoré I, Sauerborn R (2006). Housing conditions and *Plasmodium*
*falciparum* infection: protective effect of iron-sheet roofed houses. Malar J.

[23] Balami AD, Said SM, Zulkefli NAM, Bachok N, Audu B (2019). Effects of a health educational intervention on malaria knowledge, motivation, and behavioural skills: a randomized controlled trial. Malar J.

[24] Osterbauer B, Kapisi J, Bigira V, Mwangwa F, Kinara S, Kamya MR (2012). Factors associated with malaria parasitaemia, malnutrition, and anaemia among HIV-exposed and unexposed Ugandan infants: a cross-sectional survey. Malar J.

[25] Shah JA, Emina JBO, Eckert E, Ye Y (2015). Prompt access to effective malaria treatment among children under five in sub-Saharan Africa: a multi-country analysis of national household survey data. Malar J.

[26] Kass GV (1980). An exploratory technique for investigating large quantities of categorical data. J R Stat Soc Ser C.

[27] Kitsantas P, Hollander M, Li L (2006). Using classification trees to assess low birth weight outcomes. Artif Intell Med.

[28] Antipov E, Pokryshevskaya E (2010). Applying CHAID for logistic regression diagnostics and classification accuracy improvement. J Target Meas Anal Mark.

[29] ICF. The DHS Program—Malaria. The DHS Program website. https://dhsprogram.com.

[30] Obasohan PE, Walters SJ, Jacques R, Khatab K (2021). Individual and contextual factors associated with malaria among children 6–59 months in Nigeria: a multilevel mixed effect logistic model approach. Int J Environ Res Publ Health.

[31] Wanzira H, Katamba H, Okullo AE, Agaba B, Kasule M, Rubahika D (2017). Factors associated with malaria parasitaemia among children under 5 years in Uganda: a secondary data analysis of the 2014 Malaria Indicator survey dataset. Malar J.

[32] Okeke TA, Okeibunor JC (2010). Rural–urban differences in health-seeking for the treatment of childhood malaria in south-east Nigeria. Health Policy.

[33] Afoakwah C, Deng X, Onur I (2018). Malaria infection among children under-five: the use of large-scale interventions in Ghana. BMC Public Health.

[34] Ayele DG, Zewotir TT, Mwambi HG (2012). Prevalence and risk factors of malaria in Ethiopia. Malar J.

